# A Brief Account of the Diseases That Appeared on Board the Essex Prison Hulk, during the Years 1825, 26, and 27

**Published:** 1829-03

**Authors:** John Speer

**Affiliations:** Surgeon R.N. and Medical Superintendent, Essex; late Surgeon of the Castlekneck Dispensary.


### DISEASES ON BOARD THE ESSEX.

*A brief Account of the Diseases that appeared on board the Essex Prison Hulk, during the Years* 1825, 26, *and* 27. By John Speer, M.D. Surgeon R.N. and Medical Superintendent, Essex; late Surgeon of the Castlekneck Dispensary.

The Irish government having determined to station a hulk at Kingstown, considering it would be beneficial to the public, by saving the heavy expense of demurrage, as well as other expenses attendant on the shipment of prisoners for New South Wales, they accordingly stationed the Essex there, and commenced receiving prisoners in the year 1825.

The hulk became suddenly crowded, which produced disease amongst the prisoners. Catarrhal affections first made their appearance, which, generally speaking, yielded to gentle aperients, diaphoretics, and expectorants. Slight ulcers became exceedingly common, particularly on the lower extremities, chiefly created by the irons, and not unfrequently on purpose, in order to get released from their weight. The ulcers yielded, in general, to simple dressings with sulphate of copper; some to poultices; and occasionally adhesive straps were had recourse to, after the plan of Mr. Baynton. This treatment was combined with mercurial alteratives and aperients, agreeably to Mr. Abernethy's directions, to regulate the digestive organs, which were always disordered: I apprehend, from the want of proper exercise.

During the latter end of spring and beginning of summer, fever made its appearance among the prisoners. At the commencement, the disease was ushered in by a slight shivering or rigor, or probably it did not amount to either, but merely a chilliness and loss of appetite; this was succeeded by some increased heat, thirst, and headach, with a pulse not more than ninety; the bowels confined. If these symptoms were attended to at their commencement, and relief administered, the disease was in general checked or mitigated. In the cold stage, a gentle emetic very often restored reaction, and produced diaphoresis; and if afterwards followed up by a mercurial purgative and the saline effervescing draught, a crisis in eight or ten days was the result; after which, bark with wine, and a more generous diet, became necessary. If the fever advanced, the symptoms became more severe, and violent determinations or congestion of blood took place in different organs, during the reactive stage. Not unfrequently the patient became affected with violent pain in the head, throbbing of the temples, suffusion of the eyes, and constipation of the bowels. These symptoms were generally relieved by local bloodletting, shaving the head, cold applications, and mercurial purgatives. In a few cases general bloodletting was had recourse to, but the debility became so great, with only temporary relief of the local pain, that very often the application of leeches afterwards was absolutely necessary. This rendered the disease more formidable and difficult to treat, so that latterly I abandoned the practice of general bloodletting, and found much more success after local bloodletting, cold applications, and purgatives; for I believe the lancet cannot be used with that degree of freedom in the diseases of prisoners, as in those of soldiers and sailors.

It frequently occurred that petechiæ appeared, and I think I have observed it more frequently after general bloodletting. Where this appearance was unaccompanied with local congestion in the brain, or other internal viscera, the good effects of bark, wine, and the mineral acids, were very striking, and often prevented a slow fever of some weeks' duration. Notwithstanding the care that was taken to administer relief at the first onset of the disease, several cases appeared marked by very malignant symptoms, such as delirium, tongue and teeth covered with a brown crust, petechiæ, and involuntary discharges. These were treated according to circumstances: where local congestion existed, leeching and cupping were had recourse to; where the biliary and intestinal discharges were morbid, mercurial and saline purgatives were administered, with antimonials and effervescing draughts; and where the debility became very great, with subsultus tendinum, involuntary discharges, &c., wine, opium, camphor, and blisters were prescribed.

In the end of July, the greater part of the prisoners were shipped, leaving a few cases of fever.

In August we received 240 prisoners, and in a few weeks dysentery appeared: the insidious manner in which it attacked the patients induced me to consider it nothing more than a slight bowel complaint, and in many instances the individuals, for one, two, or three days, had not the slightest appearance of disease, although they were absolutely passing blood. In every case that appeared, with the exception of one or two, feces mixed with blood, or blood mixed with feces or mucus, appeared without any other bad symptom; the pulse being rather smaller than natural, the skin cool, tongue clean, and the appetite but little impaired. These symptoms generally continued some days, and the only inconvenience the patients felt was a frequent and vain desire to go to stool, without voiding any thing, or very scanty feces, which were either brown, pale coloured or whitish, or mixed with blood; but in every instance the complaint appeared to indicate functional disease of the liver. These symptoms were in general succeeded by a reaction of the system, quick pulse, tongue loaded, skin hot and dry, violent tormina and tenesmus, loss of appetite, retention of feces, and nothing passing. In one or two cases the discharges came on with tormina and feverish symptoms, accompanied with a liquid yellow solution of the feces. In no instance could scybalæ be observed, although each patient had his own night-pan. In several cases retention of urine occurred from the violence of the tenesmus, and was generally relieved by the warm bath, stupes, or enemas of starch and opium. A few patients had pain in the testicles, which continued several days, and then gradually subsided by the application of stupes. Long patches of apparently a pseudo-membranous substance were often evacuated, and appeared to me like a portion of the mucous membrane of the intestine; but I apprehend it was only coagulable lymph thrown out. As the chronic stage of the disease, and recovery, were not protracted beyond the usual time, the treatment which I generally pursued, after separating the sick, as far as circumstances would permit, consisted in the warm bath, mercurial, saline, and oily purgatives, with general and local bloodletting, as the symptoms might require; calomel with antimonial powder, and warm bath at night, followed by castor oil and tincture of rhubarb in the morning. I generally persevered in this for a few days, and, if the disease did not yield, from three to five grains of calomel, with the same of antimonial powder, and half a grain of opium, every third hour, until ptyalism was produced, in general subdued the violence of the symptoms: the stools became feculent, the tormina ceased, tenesmus less severe; and the cure was perfected by small doses of castor oil, with Dovers powder, at night; bitters and astringents, as the fever subsided.

Other cases were treated by small doses of castor oil, with from five to ten drops of tincture of opium at intervals through the day, the warm bath, a starch enema, and Dover's powder at night; which in mild cases succeeded, but was in general more protracted by this line of treatment. Small doses of sulphate of magnesia and infusion of roses, with the warm bath, and Dover's powder at bedtime, succeeded; but the benefit derived from this medicine was particularly observed when the mercurial treatment failed after ptyalism was produced.

In some obstinate cases, after the disease assumed a chronic form, when the pain had subsided, and the common astringents had failed, the acetas plumbi, in one-grain doses every third hour, with half a grain of opium, checked the disease. It may not be unworthy of remark, that the changes in the appearance of the feces in those cases where the acetate of lead was used, appeared to me so striking, that I have since doubted whether functional disease of the liver has so powerful an effect in changing the appearance of the alvine discharges; for this remedy so completely altered their appearance, from a white and often a brown colour to that of a light yellow, as to impress me with the idea that simple morbid action of the intestinal canal caused this change in the colour of the alvine discharges.

In those instances where general bloodletting was used, that decided and permanent mitigation of the pain was not observed, which I have experienced from it in inflammation of the serous membranes. I therefore conclude that inflammation of the mucous membranes are not so easily controlled by general bloodletting as that of the serous tissues.

Early in the month of October dysentery began to decline, and disappeared, when symptoms of scurvy followed. It first appeared by tenderness of the gums, sallow and unhealthy complexion, pain under the scrobiculus cordis, goose skin over the lower extremities, with slight livid patches, hardness and tightness of the lower extremities, stiffness of the joints, particularly the knees; with great despondency. As I had never observed the disease in individuals who lived on fresh diet, I felt somewhat at a loss to account for its appearance, and thought that a diminution of part of their usual proportion of vegetables might have been the cause. Many of them complained in the morning that they had not rested during the night; their breathing had been laborious, with nausea, and pain under the sternum; gums tender. When any individual was found labouring under any of the above symptoms, he was separated, and a liberal scale of diet ordered him.

In endeavouring to discover the cause of the disease, I particularly noticed it in those who had been long confined in gaols, and such as had been often disappointed in obtaining their reprieve: it therefore appeared to me that this disease was produced chiefly by long previous confinement, scanty diet, depressing passions, and crowded apartments.

My treatment at first consisted in an increased quantity of vegetables, which had not the desired effect. The nitrous vinegar, used both externally and internally, proved useless. Oranges and lemons were used with advantage; but the greatest benefit was derived from a liberal allowance of animal food, porter, and soft bread. In two prisoners the fear of transportation kept up the disease, in spite of all remedies; and it was with difficulty I could get their health so far restored as to enable them to undertake the voyage to New South Wales.

The year 1826.—During the spring quarter, a few cases of fever appeared, exhibiting no extraordinary symptoms, and yielding to the former line of treatment. In the summer months, sycosia menti and porrigo prevailed. The former attacked upwards of sixty prisoners: it appeared infectious, and as if communicated by the shaving brush; it was marked by small tumors on the chin and under lip; sometimes the cheeks, face, and scalp had their surface inflamed and thickened, and in circular clusters, healing occasionally, and followed by others; many of them the size of a pea, filled in the top with a yellow fluid like pus, often discharging this, and suppurating in the course of one night, matting the beard together, which produced a deformed appearance of the patient, and rendered shaving very difficult. In some instances the disease continued six months, alternately healing and breaking out.

My method of treatment, in the inflammatory stage, consisted of leeches, stupes, poultices, with purgatives; and, when the violence of the inflammation was subdued, alteratives, tepid baths, the unguentum nitratis hydrargyri mitius, unguentum picis, and plummer's pill. In most cases the digestive organs were much disordered, and the use of mercurial alteratives, alkalies, and decoction of bark, were all found useful. In the chronic form, the murias hydrarg. in solution proved beneficial. In a variety of instances all these remedies had but little effect, until the patients were removed from their crowded apartments to a purer air; which was generally followed by a speedy cure.

Several cases of porrigo appeared, chiefly confined to the scalp and ears: these yielded, in general, after shaving the head, to tepid ablutions of soap, oatmeal, bran, and warm water. A lotion of the sulphate of zinc, after leeches, was found useful; the hydr. unguentum nitratis was also used.

During the autumn, dysentery prevailed. Nothing particular occurred in the progress of the disease, and the mercurial plan of treatment was adopted with success.

During the year 1827, the hulk was remarked for being healthy, which I attributed to the few prisoners that remained on board; but some extraordinary diseases of the heart appeared, and one case of hypertrophy of the left ventricle occurred in a tailor, and was produced by rheumatic metastasis: the pulsation was visible in all his arteries: he lived many months, and ultimately died suddenly. Such diseases appear particularly prevalent amongst prisoners, and I believe, generally speaking, are occasioned by the depressing passions acting on, and deranging, the general circulation; and, when detected early, they may be alleviated by general and local bloodletting, purgatives, and low diet, and by removing those unfortunate individuals to their final destination.

